# The Impact of a Mindfulness Based Program on Perceived Stress, Anxiety, Depression and Sleep of Incarcerated Women

**DOI:** 10.3390/ijerph120911594

**Published:** 2015-09-16

**Authors:** Ginette G. Ferszt, Robin J. Miller, Joyce E. Hickey, Fleet Maull, Kate Crisp

**Affiliations:** 1 College of Nursing, University of Rhode Island, Kingston, RI 02881, USA; 2 School of Nursing, University of Connecticut, Storrs, CT 06269, USA; E-Mail: robin.miller@uconn.edu; 3 University of Rhode Island, College of Nursing, Kingston, RI 02881, USA; E-Mail: joyce_hickey@my.uri.edu; 4 Prison Mindfulness Institute, Providence, RI 02881, USA; E-Mails: fleet@prisonmindfulness.org (F.M.); kate@prisonmindfulness.org (K.C.)

**Keywords:** mindfulness, incarcerated women, perceived stress, anxiety, depression, sleep

## Abstract

Incarcerated women enter the prison setting with remarkable histories of trauma, mental health and substance abuse issues. Given the stress of incarceration and separation from their children, families, and significant others, it is not surprising that many women experience increased anxiety, depression, and problems with sleep. Due to these negative outcomes, it is imperative to find efficient non-pharmacological interventions. This pilot study examined the impact of a 12-week mindfulness based program on the stress, anxiety, depression and sleep of women with a total of 33 completing the study. In one group, women’s perceived stress, anxiety and depression were all significantly lower following the intervention compared to prior to the intervention. Challenges with implementing the pilot study are addressed. Despite challenges and limitations, the low-cost non-pharmacological intervention has potential for a reducing the symptoms of anxiety and depression.

## 1. Introduction

Although incarcerated women constitute approximately 7.5% of inmates, female offenders are the fastest growing population in prison today [1]. In fact, over the past 20 years, the number of women held in state and federal prisons has increased more than 6-fold, outpacing the growth of the male population [2]. In 2013, female prisoners sentenced for more than a year in state or federal prisons grew by almost 3% compared to 0.2% for men [3].

Incarcerated women are most likely to be economically marginalized, single, disproportionately women of color, and often the sole caretaker of their children [4,5,6]. Incarcerated women have higher rates of mental illness than women in the general population. Most incarcerated women report a prolonged history of physical, emotional and sexual abuse. Along with their pervasive history of victimization they often struggle with depression, anxiety and post-traumatic stress [4,6,7]. It is not surprising, therefore, that incarcerated women have a high demand for mental health services [8].

Being arrested and incarcerated is a major life event with many challenges. The numerous constraints imposed by prison life have a significant impact on the overall health and well-being of inmates. Some of the challenges and constraints include: strip searches; limitations on exercise and fresh air; prescribed and strict routine; limited visitation hours; lack of privacy (*i.e.*, mail going out and in is opened; journals can be read at any time by correctional staff); nutritional constraints (unusual mealtimes, over processed food, limited fruit and vegetables); lack of choice of health care provider; limited items purchased through canteen (shampoo, toothbrush, soap); needing to submit applications for everything including health visits, taking part in a class, *etc.*; ever changing cell mates, noise due to radios, shouting; endless waiting in corridors and lines; seeing other prisoners picked on and bullied; and/or feeling emotionally vulnerable. In addition to these challenges, the majority of women experience the burden that results from separation from their children and the effects of separation and incarceration on their children’s lives [9].

In light of their history and the constraints of prison life, women who enter correctional facilities often experience heightened anxiety, depression and problems with sleep. These symptoms can result in the costly use of the prison’s health clinics to see the physician/nurse practitioner or health care provider. Due to the negative outcomes of anxiety, depression and sleep difficulty, it is of great importance to find efficient non-pharmacological interventions to treat these distressing symptoms in correctional facilities. Cognitive behavioral groups and adjunctive treatment approaches such as music, meditation, and mindfulness may offer a cost effective, person-centered alternative to decrease anxiety, depression, improve the quantity and quality of sleep as well as decrease cost.

The purpose and specific aim of this study was to explore the impact of a 12-week Mindfulness Meditation Group on perceived stress, anxiety, depression and sleep of incarcerated women. This pilot study was conducted with one group of women in fall, 2012 and a different group of women in fall, 2013.

## 2. Methods

A pre-post non-experimental design was used to determine the impact of a mindfulness based program on perceived stress, anxiety, depression and sleep of incarcerated women.

All subjects gave their informed consent before they participated in the study. The study was conducted in accordance with the Declaration of Helsinki, and the protocol was approved by the Institutional Review Boards (Ethics Committees) of the University of Rhode Island (358202-8) and the Department of Corrections (RIDOC) where this study took place. Data was collected from August 2012 to 30 December 2013.

### 2.1. Sampling

In August, 2012, a list of potential participants was obtained from the physician, clinical social work staff and nurses. Inclusion criteria included: (1) Ability to read and speak English; (2) At least 3 months remaining in their sentence; (3) Willingness to comply with the protocol. Exclusion criteria include: Inability to give informed consent secondary to organic brain dysfunction, not having own legal guardianship, active psychosis or otherwise not able to participate or housed in segregation. For the group that was implemented in Fall 2012, a list of 27 women was given to the Principal Investigator (PI). Twenty two women agreed to participate and 18 completed the study. All participants completed the perceived stress, anxiety and depression questionnaires. A second group of participants was recruited for fall, 2013 using the same recruitment approach. A list of 26 potential participants was given to the PI, 15 agreed to participate; 15 completed the questionnaires for stress and anxiety; 13 also completed the questionnaire for depression.

### 2.2. Procedures

Once the women were identified, the PI met with each woman individually to describe the study, answer questions and review the informed consent. The PI identified herself as non-correctional staff and informed the women that study participation was completely voluntary and would not affect their health care, any privileges at the facility, or their parole. Baseline data was collected and included (a) Demographics; (b) Medical and psychiatric history; (c) Incarceration History; and (d) Medication history. One week prior to the implementation of the group, the instruments described below were administered by the research assistant (RA). The PI reviewed the instruments and the protocol for administering them to the women with the RA. These same instruments were administered by the PI or RA one week post treatment. Additionally, the initial plan was to have the same facilitator lead all 12-week group intervention programs presented, to hold constant any differences in the facilitator’s style. Circumstances required the use of additional trained facilitators after the first completed program to lead subsequent groups

### 2.3. Measures

The following measures were selected because of their reported ease of use, reliability and prior use in research with incarcerated individuals.

a. *The Perceived Stress Scale* (PSS) is the most widely used psychometric instrument for measuring the individual’s perceptions of situations appraised as stressful [10]. The 10 question items relate to health behaviors and mental health with six questions stated negatively and four stated positively [10]. Response choices for each question are on a 5- point Likert-type scale ranging from never (0) to very often (4). Positively stated questions are reverse coded before totaling the score resulting in stress level ranges from 0–40 [10]. Coefficient reliability has been reported at 0.78 [11]. The higher the score the greater the amount of perceived stress. A score of 20 or higher is considered to be indicative of a high level of stress. It is easy to use, takes about 10 min to complete and has been used in research studies with the correctional population. Multiple studies have assessed validity with other tests and found significantly strong correlation values especially with smoking behaviors, depression and anxiety [10]. Additionally, high PSS scores have been found to correlate with high cortisol levels which are a biomarker of stress [12].

b. Anxiety was measured by the *State Trait Anxiety Scale (STAI).* This is a 20-item self-report questionnaire which takes 5–10 min = to complete. It has been widely used in diverse settings. This scale has an interrater reliability score of 0.78 and a reliability coefficient of 0.91 [13]. The items ask participants to indicate how they feel right now in this moment with an easy to understand 4 point Likert scale with (1) being not at all and (4) very much so. A score of 40 suggests clinically significant symptoms for anxiety. The STAI is written at a 6th grade reading level and has been used in correctional populations.

c. Improvement in sleep was measured by *The Pittsburgh Sleep Quality Index* (PSQI). The PSQI is a self-rated questionnaire which assesses sleep quality and disturbances over a 1-month time interval. Nineteen individual items generate seven “component” scores: subjective sleep quality, sleep latency, sleep duration, habitual sleep efficiency, sleep disturbances, use of sleeping medication, and daytime dysfunction. The sum of scores for these seven components yields one global score. The PSQI is widely used in clinical research and has also been used in prison research [14]. The scale has good diagnostic sensitivity of 89.6% and specificity of 86.5% in distinguishing good and poor sleep. Internal consistency has an overall reliability coefficient of 0.83. This measure takes 10–15 min to complete.

d. *The Center for Epidemiological Study D 10* is a shorter version of the CEDS, a popular assessment tool that has been used with numerous populations including the correctional population to measure depression. A score of 15–20 indicates mild to moderate depression and a score of 20 or above indicates possible major depression. The correlation between the original and shortened scale is very high (Spearman correlation coefficient = 0.97 (*p* < 0.001) [15]. Internal consistency reliability coefficients was satisfactory (Cronbach α = 0.88). Since it is only a 10-item self-administered Likert-type scale it has great utility with the correctional population and can be completed in 5–10 min.

e. Daily Log. The women were provided with paper and pencils and asked to maintain daily logs to include descriptions of use of the CD player; any specific stressors prior to its use; length of use; specific selections on the MP3 Player and its impact on sleep, anxiety, and its perceived impact on daily functioning (involvement in activities, energy, mood).

f. Summary of study participation—a questionnaire with five open ended questions was administered on the last day (see results).

## 3. Intervention

The women participated in a 12 week program; they met for (1 1/2 h. per week) Mindfulness Based Program called *Path to Freedom.* The format for each week had the following structure: A welcome and check in for 5 min; basic mindfulness meditation for 10 min; presentation of that weeks’ topic for 15 min followed by discussion, questions and answers for 10 min. The next ten minutes was spent doing a mindfulness movement exercise and a new meditation was delivered and practiced for 20 min; Homework was reviewed for approximately 10 min; a closing meditation and final check out completed the last 10 min.

In between the group sessions, they had written homework assignments to practice skills and assignments. In addition, a CD player was given to each woman with a CD to practice relaxation and concentration meditations (Table 1). Specific meditations were given as homework each week. However, the women could listen to any of the meditations and relaxation exercises at any time. The program was delivered by a facilitator who had received training in this particular program by the Prison Mindfulness Institute and also facilitated the mindfulness based program with groups in correctional settings for a number of years prior to this study.

**Table 1:** Meditation CD.

Number	Meditation	Description and Purpose	Length of Time
1.	Gong	The CD begins with a gong that is familiar from the class. Each segment is separated by the same sound	1:37
2.	Basic Mindfulness	This meditation introduces the basic concepts of mindfulness and focusing on the breath.	4:40
3.	Self Acceptance	This meditation goes hand in hand with the workbook	3:43
4.	Be Here Now	Basic Presence Practice	2:18
5.	Breath Counting	Basic Mind Training	3:39
6.	Holding Your Seat	This meditation is used to cope with becoming triggered	9:25
7.	Deep Belly Breath	Basic Breathwork	1:23
8.	Meditation for Pain	Pain reduction	3:26
9.	Meditation for Panic	Anxiety and panic reduction	1:57
10.	Tense and Relax	Stress reduction of tensed muscles	4:41
11.	Directors Cut	The image of a director cutting after a film scene is used. The focus of this meditation Is to facilitate cutting racing thoughts.	2:51
12.	Confidence	Cultivating empowerment	2:30
13.	Noble Silence	Calming, relaxation with silence	1:55
14.	Listening	Prelude to communication and listening skills	2:03
15.	Metta	Metta means living kindness.	2:08
16.	Compassion	Cultivating Compassion for others	3:25
17.	Forgiveness	Developing Forgiveness for self and others	6:21
18.	Gong		1:37

### Description of the Path to Freedom Program

The Path to Freedom curriculum for prisoners was adapted from the Integral Peacemaker TrainingTM, a leadership training program developed by Kate Crisp and Fleet Maull for the Peacemaker Institute. The Path of Freedom curriculum is a mindfulness-based emotional intelligence (MBEI) training which also employs key elements of social emotional learning and mindfulness-based cognitive behavioral training. The program focuses on increasing participants’ resources, capacities and skills for self-awareness, mindfulness, presence, focus and attention stabilization in daily living as well as their resources, capacities and skills for self-empathy, emotion regulation, resilience, deep listening, empathic communication, problem-solving and conflict management, and forgiveness (letting go) and reconciliation in the sphere of personal and work relationships.

The Path of Freedom program also focuses on increasing participants’ resiliency, confidence and positive life outlook through direct contact with the unconditional ground of basic goodness or basic okay-ness/wellness at the core of their own being through mindfulness-awareness meditation and contemplative/reflective practices. This direct contemplative experience encourages a shift away from fear-based and often anti-social or criminal strategies for meeting needs to pro-social strategies grounded in an emerging confidence in their own basic or innate goodness, the innate goodness of others and life altogether and a greater overall sense of possibility and positive vision for their present lives and future. The program is also grounded in a holistic or whole-person, bio-psycho-social-spiritual, integral view of human development.

The Prison Mindfulness Institute (PMI), which offered the program, is a nonprofit organization incorporated in the State of Massachusetts and registered in the State of Rhode Island. The Path of Freedom curriculum is currently being taught in prisons throughout the US, Canada, Sweden, Finland, Australia, Chile and the UK. Over 600 people worldwide have completed PMI’s “Introduction to Facilitating the Path of Freedom” training. Path of Freedom is also being offered at two agencies in Boston that serve post release populations.

A growing body of research has been exploring the benefits of mindfulness on physical and psychological outcomes [16]. Although relatively little literature pertains to incarcerated populations, some literature does support the benefit of mindfulness for the general population [17]. A review of empirical studies examining the effects of mindfulness on psychological health reported a reduction of depression and anxiety [18].

Although the rapid growth in technology has opened a wide range of opportunities in the health care sector, the use of technology in correctional settings to improve health care has not yet been explored. Due to security and safety reasons, inmates are not allowed access to CD players, smart phones, iPads, *etc.* In the correctional facility where this study took place, the inmates, except for those in segregation, are allowed to purchase small radios through their purchasing system. However, sound quality is often poor and access to different channels is very limited. The Warden gave approval for the use of CD players that were purchased through a venue that is a supplier for correctional facilities.

## 4. Statistical Analysis

Using Statistical Package for the Social Sciences (SPSS) version 21, demographics (Table 2) were examined and descriptive statistics were reviewed to characterize all variables of interest and to ensure that assumptions were met for analyses. Paired-samples *t*-tests were conducted to determine if there were significant differences between the questionnaire scores one week prior and one week following the intervention.

## 5. Results

### 5.1. Demographics

#### Group I

Eighteen women participated in the first group. The women ranged in age from 20 to 53 with a mean of 34.50. They were predominantly Caucasian (61.10%) and single (72.2%). A little over half (56.6%) had been in prison before. A large percentage (83.3%) had 2 or 3 psychiatric diagnoses, primarily depression and anxiety. Forty four percent had 1 or more medical diagnosis, primarily hypertension and diabetes.

#### Group II

Fifteen women participated in the second group. Ages ranged from 22–51 with a mean age of 35.33. Forty percent of this group identified themselves as Hispanic, followed by 26.7% Caucasian. The women in this group were also primarily single (66.7%) and 66.7% had been in prison before. Forty percent had one psychiatric diagnosis, primarily depression, followed by anxiety. Twenty three percent had one or more medical diagnoses, primarily diabetes.

**Table 2:** Demographics.

Category	Variables	Fall, 2012 *n* = 18	Fall, 2013 *n* = 15
**Demographics**	Age Range M (SD)	20–53 34.50 (10.52)	22–51 35.33 (8.13)
	Race *n* (%)	Caucasian—11 (61.1%) AA—3 (16.7%) PR—2 (11.1%) SP—2 (11.1%)	Caucasian—4 (26.7%) AA—1 (6.7%) SP—6 (40.0%) Unknown—4 (26.7%)
	Marital Status *n* (%)	Married—0 (0.0%) Single—13 (72.2%) Divorced—3 (16.7%) Widowed—1 (5.6%) Missing data—1 (5.6%)	Married—2 (6.7%) Single—10 (66.7%) Divorced—1 (6.7%) Widowed—1 (6.7%) Missing data—1 (6.7%)
**Prison History**	Had been in prison before Had not been in prison before	10 (55.6%) 8 (44.4%)	10 (66.7%) 5 (33.3%)
**Psychiatric Diagnoses**	Had a psychiatric diagnosis Had 2 or 3 psychiatric diagnosis Did not have (or state) a psychiatric diagnosis	14 (77.8%) 14 (77.8%) 4 (22.2%)	6 (40.0%) 4 (26.67%) 9 (60.0%)
**Medical Diagnoses**	Had one or more medical diagnoses Did not have (or identify) a medical diagnosis	8 (44.4%) 10 (55.6%)	4 (23.5%) 13 (76.4%)

### 5.2. Data Analysis

Using Statistical Package for the Social Sciences (SPSS) version 21, descriptive statistics were reviewed to characterize all variables of interest and to ensure that assumptions were met for analyses. Paired-samples (Table 3) *t*-tests were conducted to determine if there were significant differences between the questionnaire scores one week prior and one week following the intervention There were five pieces of missing data (on two participants in the second group for the CESD-10 post-test and 3 participants for the PSQI). These participants were excluded from the respective *t*-tests due to missing data. Due to the differences in delivery of the intervention between the groups and the differences in the STAI scores between the groups (see discussion), statistical analyses were conducted separately for each group and then for the full sample.

For the first group who received the intervention in fall of 2012, women experienced significantly less stress following the intervention (*M* = 20.83, *SE* = 1.48) compared to before the intervention (*M* = 24.61, *SE* = 0.95), *t* (17) = 2.805, *p* = 0.012. For anxiety, women experienced significantly less anxiety following the intervention (*M* = 43.11, *SE* = 1.46) compared to before the intervention (*M* = 55.44, *SE* = 2.45), *t* (17) = 4.079, *p* = 0.001. On the CESD-10, women experienced significantly less depression following the intervention (*M* = 13.56, *SE* = 1.17) compared to before the intervention (*M* = 17.78, *SE* = 1.20), *t* (17) = 4.228, *p* = 0.001. However, women also had significantly lower sleep quality scores as measured on the PSQI following the intervention (*M* = 9.80, *SE* = 1.07) compared to before the intervention (*M* = 12.80, *SE* = 0.92), *t* (14) = 3.574, *p* = 0.003.

In the second group who had the intervention in fall of 2013, there were no statistically significant differences in women’s stress following the intervention (*M* = 22.93, *SE* = 1.70) compared to before the intervention (*M* = 26.67, *SE* = 1.33). Even though this is not statistically significant, the stress scores did decrease and may be of clinical significance. This finding needs further exploration. In addition, there were no statistically significant differences in women’s anxiety following the intervention (*M* = 91.13, *SE* = 3.15) compared to before the intervention (*M* = 89.80, *SE* = 2.83). The anxiety scores in this group were much higher than in the first group with a few outliers. There were no statistically significant differences in women’s reported depression following the intervention (*M* = 14.08, *SE* = 1.85) compared to before the intervention (*M* = 17.15, *SE* = 1.55). The PSQI was not used in this second group. There were significant differences between the pre-test and post-test scores on perceived stress, depression, and anxiety of all thirty-three women who were recruited between the fall of 2012 and the fall of 2013, The PSQI was used only in the first group of women, therefore reported above. Women had significantly less perceived stress, anxiety, and depression following the intervention as compared to before the intervention.

### 5.3. Open Ended Questionnaire (n = 10)

The majority of the women wanted to give verbal feedback rather than use the written questionnaire. Ten women completed the questionnaire in Group I.

1. Did you find the CD player helpful in being able to practice skills that you learned in the class? Please describe. 

*All 10 women who completed the questionnaire said “yes.” Comments included: it helped me to relax when I was stressed out; when things were going bad for me I used it to close people out and focus; the CD player was very important to me. I listened to it nightly or as needed, it kept me grounded when I was overly stressed; the CD player was helpful at allowing me to keep focused better then when I would try to meditate on my own; I found the CD helpful with the class because it went with the book and I liked the exercises on the CD; when things were going bad for me, I use it to focus; the CD helped me use mindfulness, breathing and lots more; I found the CD player to be very helpful and sometimes like a companion.*

**Table 3:** Data analysis.

Sample	Instruments	Pre-Test M (SE)	Post-Test M (SE)	Analyses
**Fall, 2012** Enrolled, *n* = 22 Completed, *n* = 18	Perceived Stress Scale (PSS)	24.61 (0.95)	20.83 (1.48)	*t* (17) = 2.805, *p* = 0.012 *
	State Trait Anxiety Scale (STAI)	55.44 (2.45)	43.11 (1.46)	*t* (17) = 4.079, *p* = 0.001 **
	The Center for Epidemiological Study D 10 (CEDS-10)	17.78 (1.20)	13.56 (1.17)	*t* (17) = 4.228, *p* = 0.001 **
	The Pittsburg Sleep Quality Index (PSQI)	12.80 (0.92)	9.80 (1.07)	*t* (14) = 3.574, *p* = 0.003 *
**Fall, 2013** Enrolled, *n* = 15 Completed, *n* = 15	Perceived Stress Scale (PSS)	26.67 (1.33)	22.93 (1.70)	*t* (14) = 2.026, *p* = 0.062
	State Trait Anxiety Scale (STAI)	89.80 (2.83)	91.13 (3.15)	*t* (14) = −0.381, *p* = 0.709
	The Center for Epidemiological Study D 10 (CEDS-10)	17.15 (1.55)	14.08 (1.85)	*t* (12) = 1.220, *p* = 0.246
	The Pittsburg Sleep Quality Index (PSQI)	N/A	N/A	N/A
**Full Sample** Enrolled, *n* = 37 Completed, *n* = 33	Perceived Stress Scale (PSS)	25.55 (0.805)	21.79 (1.11)	*t* (32) = 3.429, *p* = 0.002 **
	State Trait Anxiety Scale (STAI)	71.06 (3.53)	64.94 (4.52)	*t* (32) = 2.395, *p* = 0.023 *
	The Center for Epidemiological Study D 10 (CEDS-10)	17.52 (0.94)	13.77 (1.02)	*t* (30) = 3.155, *p* = 0.004 **
	The Pittsburg Sleep Quality Index (PSQI)	Utilized with the first group only. See results above		

* *p* < 0.05; ** *p* < 0.01.

2. What if anything did you particularly like about the CD?

*I knew that I might struggle and it helped me focus back on my breath, I liked the length and the different kinds; Being able to listen when I was feeling stressed; I used it whenever I could; The CD was very soothing; it helped me to stay focused; Talking me through the meditation; it helped me to relax at night; deep breathing exercises;*

3. What if anything did you dislike about the CD?

*I would have liked longer versions; I did not dislike anything; Nothing (n = 3); I wish the CD player was smaller; I sometimes didn’t want to hear the same things over and over; I would have enjoyed a second track; the earphones hurt my ears; the sound of the woman’s voice.*

4. Do you have any suggestions for what we might change about the CD?

*Not really (n = 6); add music to the background; use different voices; add more native sounds like waves; add ocean sounds, birds chirping.*

5. If you did find using the CD player and CD helpful, can you tell us approximately how often you used it and how it helped?

*I used it every night to fall asleep (n = 2); daily to help me calm down; about 4 times a week for about 2 h…it helped me stay grounded, helped me stay focused and in the moment; everyday…oh boy did it help…I am very dependent on it and scared to see how I will do without it; every day to decrease my stress; I used it a lot to help with my stress; all the meditations and relaxations were helpful to me. Now I have more tools to use when I get stressed; I used it every other day; I used it daily especially when I started feeling anxious or overwhelmed. It would help me to calm down and get grounded.*

## 6. Discussion

There were major differences between Group I and Group II in terms of delivering the intervention which impacted this study. In Group I, who had a consistent facilitator, the women experienced significantly less perceived stress, anxiety, and depression following the intervention compared to before the intervention. It is possible that positive findings could be attributable to external factors, such as release date from prison. The trained interventionist for this group had provided this program in other correctional facilities. Based on informal feedback from the women who participated, the interventionist was clear, well organized and well-paced. She had a positive interpersonal style and was easily able to connect with the women. Unfortunately due to life events, this facilitator was not able to continue with this program as was initially planned. Due to time constraints it was not possible to have one specific facilitator for the second group, therefore three different facilitators were used to deliver the program.

Women in the first group had significantly lower sleep quality scores as measured on the PSQI following the intervention compared to before the intervention. This may be due to environmental changes in the prison system over the course of the study such as noise, lack of activity, extreme temperatures, and/or boredom [14]. In addition, the ingestion of stimulating beverages such as coffee were not measured which could have an effect on the women’s quality of sleep.

Group II had different facilitators from week to week. These instructors were also trained by the Prison Mindfulness Institute, however their styles were different as one might expect. Although the Principal Investigator was aware of this issue there was no alternative in program delivery at this time. Additionally, the final open ended questionnaires were not distributed to the second group and therefore that data was lost.

Even though there were no statistically significant differences between stress and depression scores before and after the intervention in this second group of women, there were still decreases in the mean scores. The lack of statistical significance between perceived stress and depression scores before and after the intervention may have been due to the great deal of turmoil that occurred in the correctional facility during the second group’s participation in the study. There were changes in the women’s assigned rooms as well reassignment of their cellmates. This environmental change led to a great deal of frustration and “shutting down” emotionally by the women. It is not possible to determine how much impact these factors had on this second group. However, it is important to note that there was still a decrease in the stress and depression scores of this group despite the numerous challenges.

In examining the anxiety scores of the second group of women, the pre- and post-intervention scores were significantly higher compared to the first group of women. The mean pre-intervention anxiety score for Group I was 55.44 compared to Group II (*M* = 89.80) (*t* (32) = −9.234, *p* < 0.0001). The post-intervention anxiety score for Group I was 43.11 compared to a mean of 91.13 in Group II (*t* (32) = −14.612, *p* < 0.0001). In addition, there were several outliers in the second group which increased the mean score. The Pittsburgh Sleep Quality Index was not used in the second group since it proved to have a high response burden; the women found it to be too difficult to complete even with additional instructions. The first pre-test administration of this instrument, the researchers decided to remove it from the study. Completion of the daily logs to include descriptions of use of the CD player; any specific stressors prior to its use; length of use; specific selections on the MP3 Player and its impact on sleep, anxiety; and its perceived impact on daily functioning (involvement in activities, energy, mood), proved to be ineffective as the women were not consistently completing them. Therefore, in order to obtain qualitative data, a 5-question open-ended questionnaire was given to the women in group I on the last day of their program. Results are presented above.

It is important to note that the PI continues to receive positive verbal feedback from the women about the program and the use of the CD players. A number of women who did not participate in the study asked if they could receive the CD as they have heard that their cellmates sleep better, use their CD player to decrease their anger and help deal with painful ongoing issues with the multiple losses that they experience.

### 6.1. Challenges

A number of researchers have discussed the methodological challenges experienced in conducting research in correctional facilities [19,20]. The following challenges confronted the researchers in the implementation of this study: (1) Although participants had release dates in place, some inmates were released earlier than expected or transferred to the minimum facility; (2) Maintaining a consistent and appropriate space for this type of group was an ongoing issue. A quiet space that does not allow for intrusions during the class is absolutely necessary; (3) A consistent interventionist is necessary in order to be certain that the program is delivered the same to everyone. Life circumstances interfered with a consistent interventionist in the second group and therefore violated intervention fidelity; (4) Finding a quiet room where participants can complete pre and post measures was extremely difficult in this setting. Security concerns and changing schedules interfered with data collection at times during the second group.

The prison setting is filled with unpredictability and events that can have a profound, positive or negative impact on a participant which can affect scoring of instruments. News of a families’ serious illness or death, failure of a visitor to come, being denied parole, the release of a close roommate, a persistent toothache, a change in a correctional officer’s schedule or assignment are only a few examples of events that can affect data collection.

### 6.2. Limitations

One limitation of this study was the convenient, small sample size of the first group however this was a pilot study and the size was appropriate for the statistical analyses conducted. The sample was representative of the target study population and was large enough to provide useful information about the aspects that are being assessed for feasibility [21]. In addition, lack of a controlled group does limit the interpretation of the findings. Unfortunately, there were problems with the delivery of the intervention in the second group but the PI was able to confirm the importance of a consistent facilitator who was clear, well organized, and well-paced, with a positive interpersonal style.

## 7. Conclusions

Despite the challenges and limitations experienced in conducting this research study, the results are promising. During the first group when the correctional facility was more stable and there was a consistent facilitator of the intervention, the women exhibited lower scores on stress, anxiety and depression following the intervention. The cost of the CD player and CDs are approximately $42 per person. The cost of the facilitator can range from $50–$100 per hour. This low cost treatment approach offers potential utility for use in correctional settings and may lead to cost savings in treating stress, anxiety and depression in this population. Future research using randomized controlled trials will be important to provide evidence of the efficacy of this intervention. However, the availability of a quiet, consistent space without interruptions to deliver the program, and a consistent facilitator will be essential in determining the feasibility of future research with this treatment approach. It will also be important to consider other treatment modalities as well.
